# Reliability Study of Magnesium Oxychloride-Coated Reinforced Concrete Based on Gumbel Distribution

**DOI:** 10.3390/ma16062521

**Published:** 2023-03-22

**Authors:** Yuanke Li, Hongxia Qiao, An Yang

**Affiliations:** 1 School of Civil Engineering, Lanzhou University of Technology, Lanzhou 730050, China; 2 Gansu Advanced Civil Engineering Materials Engineering Research Center, Lanzhou 730050, China

**Keywords:** accelerated corrosion, relative dynamic elastic modulus, gumbel distribution, life prediction

## Abstract

The constant current accelerated corrosion test was used to study the durability of magnesium oxychloride-coated reinforced concrete (MOCRC) in order to solve the problem of MOCRC’s durability. The relative dynamic elastic modulus was utilized as the failure threshold to evaluate the concrete durability, and the collected life data of concrete under different cover thickness were acquired. On the basis of the Gumbel distribution, the probability analysis can be used to study and foretell the life data. The results show that when the durability is evaluated by the relative mass and the relative dynamic modulus of elasticity, the durability of MOCRC with a larger protection layer thickness is better; the relative dynamic modulus of elasticity can better reflect the durability change in MOCRC than the relative mass. When the Gumbel distribution is used for durability analysis, the calculated value of the model and the life data have a relatively high degree of fit, which can provide a reference basis for the durability evaluation of concrete.

## 1. Introduction

Magnesium oxychloride cement is an air-hardening cementitious material, which is prepared by mixing a magnesium oxychloride solution of certain concentration with light-burned magnesium oxide. Because of the characteristics of its cementitious material, MOCRC itself has a certain resistance to brine, so it is suitable for the saline soil environment [1]. However, due to the corrosion of steel bars by Cl^−^ contained in raw materials, the application of MOCRC in load-bearing structures is limited [2]. The coating technology can slow down the corrosion rate of steel bars in MOCRC [3,4]. However, due to the different actual service environment of concrete, the service life of coated steel bars is quite different, so it is necessary to study the durability of coated reinforced concrete.

By now, studies on the durability of concrete mainly include the natural exposure test and the accelerated corrosion test [5,6,7], but the natural exposure test is time consuming and has many influencing factors; therefore, it is not conducive to the research. Most scholars choose the method of accelerated corrosion to study the durability of concrete [8,9]. The commonly used methods of accelerated corrosion include the full immersion method, half immersion method, veneer method, and alternate dry and wet method [10,11,12,13]. Although the above methods can quickly achieve the effect of reinforcement corrosion, the severe hypoxia during the test is not consistent with the corrosion of reinforcement in the natural environment. Feng [14] used wet salt sand instead of a corrosive salt solution as the electrolyte to study. The results showed that compared with ordinary salt solution, the electrified accelerated corrosion test using wet salt sand as the electrolyte can better simulate the corrosion of reinforcement in the natural environment. In this paper, saline soil is used as the electrolyte to simulate the corrosion of MOCRC in the natural environment.

Scholars use different experimental methods to simulate the degradation process of concrete. Yang [15] proposed the calculation model and quantitative analysis method of the durability of a concrete structure in the marine environment. Shi [16] further modified the existing strength attenuation model based on the freeze-thaw cycle test results; the attenuation model of PRGRC compressive strength under the freeze-thaw cycle is obtained. Kousa [17] concluded that the coupling effect has a synergistic effect on the durability degradation of concrete by simulating the durability degradation of concrete in the natural environment; the overall degradation process of concrete durability can be predicted quantitatively. Alrayes [18] presented numerical simulations of mixed-mode crack propagation in concrete using the scaled boundary finite element method. However, the deterioration of concrete durability is caused by the coupling of many factors, and it is a constantly changing process in the service process of concrete structures. Therefore, using the fixed model to predict the service life of concrete has some errors.

In order to avoid errors caused by the fixed model prediction, some scholars began to use the probability method to predict the service life of concrete. Ryan and O’Connor [19] used the probability method to obtain the life distribution function of self-compacting concrete, so as to evaluate its reliability. Qiao [20] studied the three-parameter Weibull distribution to predict the accelerated life of concrete. The study showed that its fitting degree and accuracy were good. Compared with the above methods, the Gumbel distribution function can obtain a more accurate failure analysis and prediction for small sample data, so it is widely used in reliability engineering, such as meteorology and hydrology [21], pipeline corrosion [22], concrete pitting depth [23], and so on. However, there are few studies on the application of the Gumbel distribution for concrete life prediction.

To sum up, this paper uses the power-on accelerated corrosion test to study the durability degradation law of MOCRC; carries out a quality test, ultrasonic test, and SEM test on MOCRC; and uses the Gumbel distribution to model based on the measured value of life data to realize the durability prediction of concrete structures during service.

## 2. Experiment

### 2.1. Experimental Materials

The mix ratio used in the test is the better MOCRC mix ratio obtained from the previous test of the research group, and the raw materials are mainly composed of magnesium oxide (MgO), magnesium chloride (MgCl_2_), water reducer, water resistant agent, fly ash, gravel, sand, and steel bars. The chemical composition of MgO, MgCl_2_, and fly ash is shown in Table 1, Table 2 and Table 3, and the performance indicators of sand and stone are shown in Table 4 and Table 5. The water reducer is a K-naphthalene series superplasticizer. The water-resistant agent is produced by Tianjin Baishi Chemical Co., Ltd. (Tianjin, China), with chromaticity units ≤ 25 and phosphoric acid content > 85%. The water is tap water from the Lanzhou area. The steel bars are all HRB400, and the diameter is 12 mm. The coating is modified epoxy resin coating, which is mainly composed of ultra-fine flake zinc and ultra-fine flake aluminum. The coated steel bar is made in the factory, and the coating method is electrostatic spraying. The average thickness of the coating is 80 μm. The mix proportion of MOCRC is shown in Table 6.

### 2.2. Test Scheme

**Figure 1:**
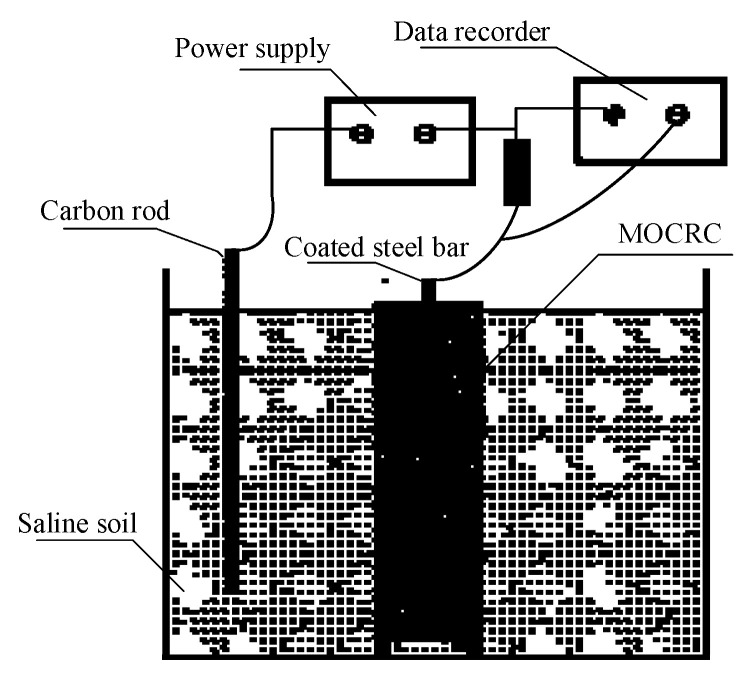
Schematic chart of electrified accelerated corrosion system.

The MOCRC specimen is prepared in accordance with the standard of “Test method for long-term performance and durability of ordinary concrete”. At the same time, in order to better observe the rust expansion and cracking of MOCRC in the process of electrified accelerated corrosion, the size of the mold is 100 mm × 100 mm × 400 mm, and the protective layer’s thickness is 25 mm for group A and 44 mm for group B. After the specimen was formed for one day, the mold was taken out and cured in the standard environment for 28 days. The cured MOCRC specimens were tested by relative dynamic elastic modulus and mass, and then the electrified accelerated corrosion test was carried out. The schematic chart of the accelerated corrosion system of the coated steel bar during electrification acceleration is shown in Figure 1.

The constant current accelerated corrosion mode is adopted in the electrified accelerated test, and the corrosion density by now is set to 100 μA/cm^2^; the power supply is the DC voltage stabilized one. In the test of the accelerated corrosion, the anode of the DC power supply is connected with the coated steel bar in MOCRC, and the cathode is connected with the carbon rod; the average resistivity of the carbon rod is 47.30 mΩ. In this experiment, the saline soil in the Golmud area was used as the electrolyte, and the results of the corrosive ion content analysis are shown in Table 7. In the process of accelerating corrosion by constant current electrification, the relative dynamic elastic modulus and mass should be tested every 3 days.

In this paper, the relative dynamic modulus of elasticity and relative mass are used to evaluate the durability of MOCRC [24]. The relative mass evaluation parameter is expressed as follows [24]:

(1) $$M_{r}=\frac{M_{t}}{M_{0}}$$

*M*_0_ is the initial mass of MOCRC before the test; *M_t_* represents the mass of MOCRC in different acceleration stages.

The calculation formula of the relative quality evaluation parameters is as follows [24]:

(2) $$\omega_{1}=\frac{M_{r}-0.95}{0.05}$$

The relative dynamic modulus of elasticity can be expressed as [24]:

(3) $$E_{r}=\frac{E_{t}}{E_{0}}=\frac{v_{t}}{v_{0}}$$

*E*_0_ is the initial dynamic elastic modulus before the test, *E_t_* is the dynamic elastic modulus at *t*; *v*_0_ is the initial ultrasonic velocity before the test, and *v_t_* is the ultrasonic velocity of the test piece at t. Then the evaluation parameters of the relative dynamic elastic module is as follows [24]:

(4) $$\omega_{2}=\frac{E_{r}-0.6}{0.4}$$

*ω* < 0, MOCRC reaches the destruction standard; 0 ≤ *ω* < 1, the durability of MOCRC decreased, but the failure criteria were not met; *ω* ≥ 1, MOCRC was in good condition.

## 3. Test Results and Data Analysis

### 3.1. Durability Change Rule of MOCRC

**Figure 2:**
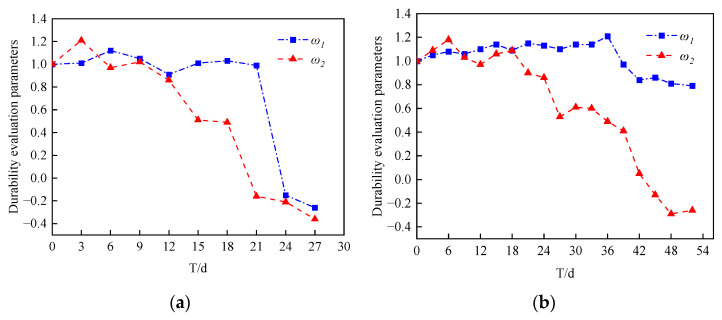
Durability evaluation parameters of MOCRC. (**a**) Group A. (**b**) Group B.

The change curve of the relative quality evaluation parameter *ω*_1_ and the relative dynamic modulus of the elasticity evaluation parameter *ω*_2_ with time of the MOCRC under accelerated corrosion is shown in Figure 2.

According to Figure 2a, in the early stage of the accelerated corrosion test, the MOCRC durability evaluation parameters ω_1_ and ω_2_ with a protective layer thickness of 25 mm were greater than 1, indicating that the MOCRC durability was good at the early stage of the test. After 12 days of the test, the durability evaluation parameters ω_1_ and ω_2_ of MOCRC decreased rapidly and were always less than 1, indicating that MOCRC was in a state of durability deterioration. On day 21, the ω_2_ of MOCRC was −0.16, which reached the failure threshold. At this time, the ω_1_ was 0.99, which did not meet the failure criteria. According to Figure 2b, at the initial stage of the accelerated corrosion test, the MOCRC durability evaluation parameters ω_1_ and ω_2_ with a protective layer thickness of 44 mm were greater than 1. On day 9 to day 39, the ω_1_ of MOCRC showed an overall upward trend, while for ω_2_, although there was an upward trend on day 12 to day 18, it showed a fluctuating downward trend. On day 45, the ω_2_ of MOCRC was −0.13, which reached the failure threshold. At this time, the ω_1_ was 0.79, which did not reach the failure threshold. Compared with Figure 2a,b, the durability degradation rate of MOCRC is related to the thickness of the protective layer. When the thickness of the protective layer is large, the durability degradation rate is slow.

According to Figure 2, MOCRC with a protective layer thickness of 25 mm and 44 mm is the relative dynamic modulus of elasticity evaluation standard ω_2_ that reaches the failure threshold first. This is because MOCRC is buried in saline soil, and some substances will enter into MOCRC during power-on acceleration, which will improve the quality of MOCRC. At this time, due to the corrosion of reinforcement, rust expansion cracks have been generated in MOCRC, so the relative dynamic elastic modulus of MOCRC is reduced. That is, the durability of MOCRC can be more effectively evaluated when the relative dynamic modulus of elasticity is taken as the evaluation parameter. This result is the same as that of the literature [25,26,27,28].

### 3.2. SEM Test Results of MOCRC

During the process of power-on accelerated corrosion of MOCRC, the deterioration of the macro performance of the test piece will inevitably lead to the change in its internal microstructure, and the change in microstructure will also reflect the durability of MOCRC. In order to better reflect the deterioration of the durability of the specimens, the specimens with different protective layer thickness were analyzed by SEM (scanning electron microscope), and the durability of the magnesium cement concrete structure during the accelerated corrosion process was further analyzed from the microscopic perspective.

**Figure 3:**
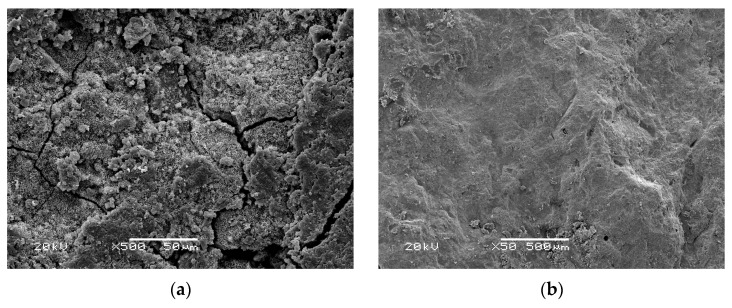
SEM of MOCRC. (**a**) Group A. (**b**) Group B.

After 20 days of the accelerated corrosion test, the MOCRC was subjected to a SEM test. The SEM test results of different protective layer thickness are shown in Figure 3.

According to Figure 3, the MOCRC with a protective layer thickness of 25 mm is severely corroded after 20 days of accelerated corrosion, which is mainly manifested by cracks and flaky corrosion products in the microstructure. The MOCRC with a protective layer of 44 mm has a relatively light corrosion after 20 days of accelerated corrosion, and its microstructure is relatively dense and uniform, without obvious corrosion products. The degree of durability deterioration of MOCRC under electrified accelerated corrosion is related to the thickness of the protective layer, that is, the smaller the thickness of the protective layer of MOCRC, the more serious the degree of durability deterioration. This result is the same as the macro performance of the previous section.

### 3.3. Life Data of MOCRC

**Figure 4:**
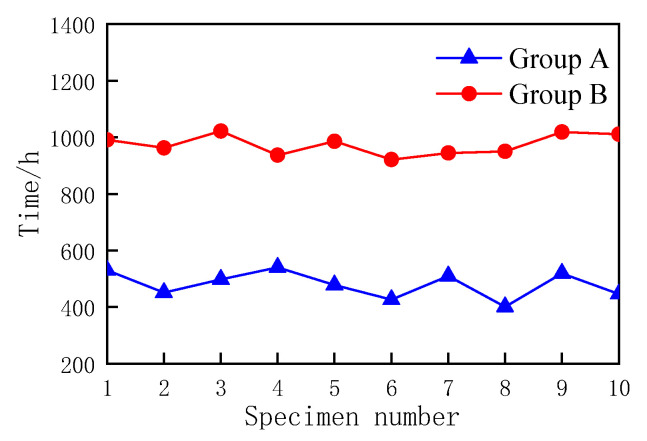
Accelerated life of MOCRC specimens with different thickness of protective layer.

According to the conclusion in Section 3.1, the life of MOCRC is determined by the relative dynamic modulus of elasticity without evaluation parameters. In this accelerated corrosion test, there are 10 specimens for each protective layer thickness. When the relative dynamic modulus of elasticity evaluation parameter is 0, the accelerated corrosion life data of MOCRC are shown in Figure 4.

## 4. Durability Analysis of MOCRC Based on Gumbel Distribution

### 4.1. Gumbel Distribution Basic Model

The Gumbel distribution is applied in many fields, such as annual maximum wave height, annual maximum temperature difference, etc. If the accelerated corrosion life data obey the Gumbel distribution, its cumulative failure distribution function, reliability function, and probability density function are respectively shown in Equations (5)–(7) [21]:

(5) $$F\left(t\right)=1-\exp\left[-\exp(\frac{t-\mu }{\sigma })\right]$$

(6) $$R\left(t\right)=\exp\left[-\exp(\frac{t-\mu }{\sigma })\right]$$

(7) $$f\left(t\right)=\frac{1}{\sigma }\exp\left[\frac{t-\mu }{\sigma }-\exp\left(\frac{t-\mu }{\sigma }\right)\right]$$

In the above equations, *t* represents time, *μ* is the location parameter, and *σ* is the scale parameter.

### 4.2. Gumbel Extremum Distribution Fit Test and Parameter Estimation

After the test data are fitted with a specific distribution, it is necessary to test the fitness of the selected distribution in order to evaluate whether the selected distribution is appropriate or not. If it passes the fitting degree test, the test data can be modeled with the selected distribution and the relevant parameters can be calculated; if it fails the test, it indicates that the distribution cannot be used to fit the test data. First of all, draw a probability diagram to make a preliminary study of the concrete accelerated life data, and judge the quality of the fitting data according to the discreteness of the sample points of the test data and the transformed distribution fitting line.

To obtain a relative accurate degree of fit further, the A–D test is used, and the significance level is 0.5. If the *p* value in the A–D test is greater than 0.05, it is considered to pass the distribution test. At the same time, on the basis of the accelerated life test data of magnesium cement concrete specimens, the parameters of the accelerated corrosion life distribution of Group A and Group B specimens can be estimated by using the maximum likelihood estimation built-in in Minitab17.1 statistical analysis software.

The principle of maximum likelihood estimation is as follows: let *t*_1_, *t*_2_, …, *t_n_* be a sample value corresponding to sample *T*_1_, *T*_2_, …, *T_n_*, the likelihood function of the Gumbel distribution is [21]:

(8) $$L(\sigma ,\mu )=L(t_{1},t_{2},\ldots ,t_{n};\sigma ,\mu )=\prod_{i=1}^{n}\frac{1}{\sigma }\exp\left[\frac{t_{i}-\mu }{\sigma }-\exp\left(\frac{t_{i}-\mu }{\sigma }\right)\right]$$

(9) $$L(t_{1},t_{2},\ldots ,t_{n};\hat{\sigma },\hat{\mu })=\max\left\{\prod_{i=1}^{n}\frac{1}{\sigma }\exp\left[\frac{t_{i}-\mu }{\sigma }-\exp\left(\frac{t_{i}-\mu }{\sigma }\right)\right]\right\}$$

Then $\hat{\sigma }\left(t_{1},t_{2},\ldots ,t_{n}\right)$ and $\hat{\mu }\left(t_{1},t_{2},\ldots ,t_{n}\right)$ are the maximum likelihood estimators of *σ* and *μ*, and the solution equation is as follows:

(10) $$\frac{d\ln L(\sigma ,\mu )}{d\sigma }=\frac{\ln\left(\prod_{i=1}^{n}\frac{1}{\sigma }\exp\left[\frac{t_{i}-\mu }{\sigma }-\exp\left(\frac{t_{i}-\mu }{\sigma }\right)\right]\right)}{d\sigma }=0$$

(11) $$\frac{d\ln L(\sigma ,\mu )}{d\mu }=\frac{\ln\left(\prod_{i=1}^{n}\frac{1}{\sigma }\exp\left[\frac{t_{i}-\mu }{\sigma }-\exp\left(\frac{t_{i}-\mu }{\sigma }\right)\right]\right)}{d\mu }=0$$

### 4.3. Gumbel Distribution Fit Test Results

**Figure 5:**
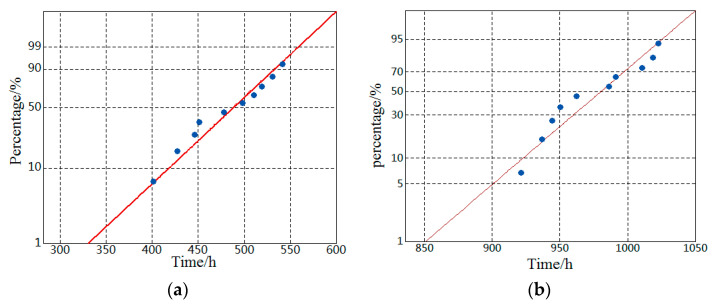
Fitting probability diagram of accelerated life data of MOCRC. (**a**) Group A. (**b**) Group B.

Using Minitab statistical analysis software, the distribution probability map is drawn to test the accelerated life data. The specific fitting diagram is shown in Figure 5.

The life data of MOCRC obtained from the electrified accelerated test do not rely on any distribution. The fitting line of the transformed distribution is different because of the different distribution, so the probability graph can be used to evaluate the fitting degree between the Gumbel extremum distribution and the electrified accelerated life data. It can be obtained through Figure 5; the electrified accelerated life data of MOCRC are close to the fitting line; the sample points are all near the fitting straight line. It shows that the Gumbel distribution has a good fitting degree for the constant current accelerated corrosion life data of MOCRC. To assess the fitting degree of the Gumbel extremum distribution more accurately, a further test is needed.

The accelerated corrosion life of Group A and Group B specimens are tested by the Gumbel distribution A–D (Anderson–Darling) test and parameter estimation. The *p* value in the A–D test obtained by Minitab17.1 statistical analysis software is evaluated for whether it obeyed the Gumbel extremum distribution or not. The results are shown in Table 8.

According to the A–D test, the A–D values of Group A and B are all less than 0.5, which indicates that the fitting line of the Gumbel distribution has a high fitting degree with the distribution of test data sample points. Additionally, the P values are more than 0.05; therefore, it can be concluded that the constant current accelerated corrosion life of MOCRC specimens with different thickness of the protective layer obeys the Gumbel distribution.

### 4.4. Prediction and Evaluation of Accelerated Corrosion Life of MOCRC

**Figure 6:**
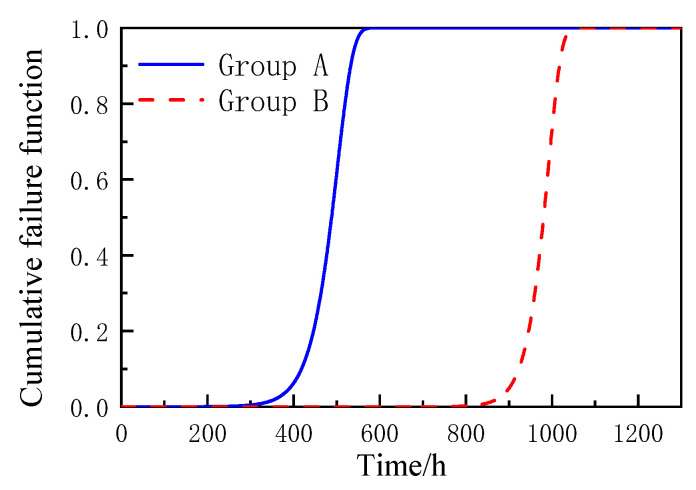
The cumulative failure function diagram of MOCRC.

**Figure 7:**
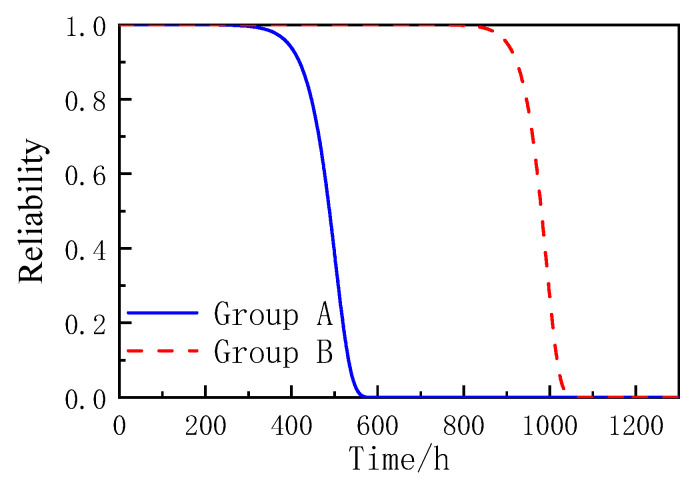
The reliability function diagram of MOCRC.

**Figure 8:**
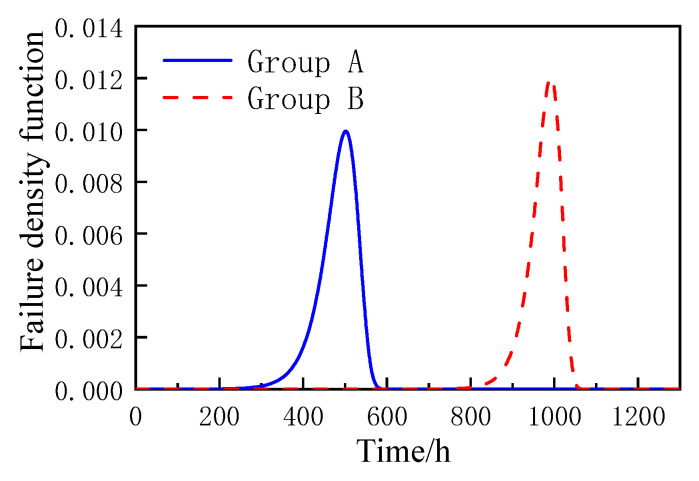
The probability density function of MOCRC.

Substitute the position and scale parameters into Equations (5)–(7), and the accelerated life cumulative failure function *F*(*t*), reliability function *R*(*t*), and failure density function *f*(*t*) of coated reinforced cement magnesium cement concrete specimens based on the Gumbel distribution can be obtained as shown in the formula, and the curve diagram is shown in Figure 6, Figure 7 and Figure 8.

According to Figure 6, Figure 7 and Figure 8, the Gumbel distribution profile of MOCRC specimens under the accelerated corrosion action of electrification varies with the thickness of the protective layer of concrete reinforcement, and the thickness of the protective layer of reinforcement has a great impact on the accelerated life of the specimens. As can be seen from the reliability function curve, the reliability of the accelerated corrosion life of the specimen decreases with the prolonging of the constant current electrification time. Among them, the Group A specimen has a large change after 300 h of accelerated corrosion, and it can be judged as a complete failure after 560 h. After 800 h of accelerated corrosion, the variation range of the Group B specimen is large, and it can be judged as a complete failure after 1050 h.

In order to better assess the Gumbel extremum distribution on Group A and B specimens’ accelerated life prediction, in combination with Minitab17.1 statistical analysis software to analyze the distribution profile, we calculated the failure times and confidence intervals for different thickness of protective layer of concrete specimen at failure rates of 30% and 70%. The specific data are shown in Table 9.

From Table 9, it is shown that the failure time of H25 specimens based on the Gumbel distribution is 463.525 and 509.762, respectively, when the accelerated life failure rate of Group A specimens is 30% and 70%, respectively. The failure time of specimens in Group B is 960.138 h and 997.147 h, respectively. According to the calculation, when the failure rate is 70%, the time for the failure of the accelerated life of the concrete specimens is basically consistent with the mean value of the sample points of the test data, indicating that the method of predicting the accelerated life of MOCRC under constant current based on the Gumbel distribution is feasible, thus providing a new method for the durability assessment of concrete structures in actual service.

## 5. Conclusions

This paper is based on the electrified accelerated corrosion test of MOCRC; the dynamic elastic modulus is used as the failure criterion of concrete, and the Gumbel extremum distribution model is used to obtain the life reliability function of MOCRC electrified accelerated corrosion under different protective layer thickness. We conclude the following:

1. The durability of MOCRC specimens with different protective layer thickness is different under the accelerated corrosion effect of electrification, and the accelerated corrosion life of Group B specimens is greater than that of Group A specimens.
2. Using the electrified accelerated corrosion test, the durability of MOCRC can be better evaluated using the relative dynamic modulus of elasticity rather than using relative mass as a parameter.
3. The reliability of MOCRC obtained by the Gumbel distribution is basically consistent with the actual test data, indicating that it is effective to use the Gumbel distribution to predict the reliability of MOCRC.

## Figures and Tables

**Table 1:** Magnesium oxide chemical composition.

MgO	Active MgO	CaO	SiO_2_	Loss of Ignition	Others
90	48.6	1.1	3.2	3.8	1.9

**Table 2:** Magnesium chloride chemical composition.

MgCl_2_·6H_2_O	SO_4_^−^	K^+^ + Na^+^	CaCl_2_	Other
96	0.4	1.2	0.4	2.0

**Table 3:** Grade I fly ash chemical composition (%).

Mg	Ca	Fe_2_O_3_	Al_2_O	SO	Loss of Ignition	SiO
1.19	5.3	9.43	20.93	0.41	3.26	54.32

**Table 4:** Performance index of sand.

Sediment Percentage/%	Clay Lump/%	Apparent Density/(kg/m^3^)	Loose Packing Density/(kg/m^3^)	Compact Packing Density/(kg/m^3^)	Voidage/%	Moisture Content/%
2.396	0.15	2610	1600	1640	38.889	2.737

**Table 5:** Performance index of gravel.

Sediment Percentage/%	Clay Lump/%	Apparent Density/(kg/m^3^)	Loose Packing Density/(kg/m^3^)	Compact Packing Density/(kg/m^3^)	Voidage/%	Moisture Content/%
0.5	0.2	2780.0	1520.0	1640.0	45.3	0.3

**Table 6:** Mix proportion of magnesium oxychloride cement reinforced concrete.

MgO/kg	Water Reducer/kg	Fly Ash/kg	Water Resistant Agent/kg	Sand/kg	Pebbles/kg	MgCl_2_/kg	Water/kg
388.96	16.02	68.64	4.58	625.00	1162.00	147.81	135.59

**Table 7:** Soil quality analysis of saline soil.

	Anion Content/mg·kg^−1^				Cation Content/mg·kg^−1^			Total Amount/%
Project	CO_3_^2−^	HCO_3_^−^	SO_4_^2−^	Cl^−^	Ca^2+^	Mg^2+^	Na^+^ + K^+^	/
	59	181	15,646	81,016	5840	379	22,887	12.603

**Table 8:** Test of fitting degree of test piece and result of parameter estimation.

Specimen	A–D	P	Location Parameter	Scale Parameter
Group A	0.247	>0.25	501.7	36.97
Group B	0.389	>0.25	991.6	30.50

**Table 9:** The failure time corresponding to the accelerated life failure rate of MOCRC.

Specimen	Failure Rate/%	Failure Time/h	95% Confidence Interval
H25	30	463.525	(428.076, 498.974)
	70	508.792	(485.327, 532.257)
H44	30	960.138	(931.153, 989.123)
	70	997.147	(977.967, 1016.53)

## Data Availability

The data presented in this study are available upon request from the corresponding author.
